# Identification of Three Prognosis-Related Differentially Expressed lncRNAs Driven by Copy Number Variation in Thyroid Cancer

**DOI:** 10.1155/2022/9203796

**Published:** 2022-05-20

**Authors:** Jinyi Tian, Bin Luo

**Affiliations:** Department of General Surgery, Beijing Tsinghua Changgung Hospital, School of Clinical Medicine, Tsinghua University, 168 Litang Road, Changping District, Beijing, China

## Abstract

Thyroid cancer as the malignant tumor with the highest incidence in the endocrine system also shows a fast growth and development. In this work, we developed a new method to identify copy number variation– (CNV–) driven differentially expressed lncRNAs in thyroid cancer for predicting cancer prognosis. The data of RNA sequencing, CNV, methylation, mutation, and clinical details of thyroid cancer were obtained from the Cancer Genome Atlas database (TCGA). Molecular subtypes were clustered by iClusterPlus. Weighted gene co-expression network analysis (WGCNA) was employed to show co-expression modules. DEseq2 was conducted to identify protein coding genes (PCGs) and differentially expressed lncRNAs. CNV was detected using GISTIC 2.0. Three molecular subtypes were identified, and 68 differentially expressed lncRNAs (DElncRNAs) related to cancer were found among different molecular subtypes. CNV of FOXD2-AS1, FAM181A-AS1, and RNF157-AS1 was associated with overall survival and was involved in cancer-related pathways. These three DElncRNAs discovered based on CNV could serve as prognostic biomarkers to predict prognosis for thyroid cancer and new targets to explore molecular drugs.

## 1. Introduction

Thyroid cancer, which is a common thyroid malignancy, can be classified into medullary cancer, papillary cancer, undifferentiated cancer, and follicular cancer by pathological classification. According to the statistics of cancer in 2018, 567,000 new cases of thyroid cancer occurred worldwide, accounting for 3.1% of all new cancer cases of the year. Follicular carcinoma and papillary thyroid carcinoma composed the majority of all thyroid cancer [1, 2]. Although most patients have a positive prognosis and survival after treatment, lymph node metastasis is common as well, and patients are prone to develop into advanced cancer and recurrence. Therefore, it is important to study its specific pathogenesis to guide new treatments [3]. Sufficient evidence indicated that the development and progression of papillary thyroid carcinoma is not only affected by environmental and genetic susceptibility factors, but also by genetic changes [4].

Long noncoding RNAs (lncRNAs), a group of RNAs with a length over than 200 nucleotides, play a critical role in gene expression regulation. In recent years, a number of studies showed that lncRNAs are closely involved in the development and progression of cancers [5–7]. LncRNAs have been considered promising biomarkers for cancer detection and prognosis follow-up [8–10]. For example, the expression level of H19 is promoted in multiple cancers, for example, cervical cancer, breast cancer, and bladder cancer. H19 expression is also higher in thyroid cancer tissue than in normal tissue, which may promote the migration of thyroid cancer cells [11, 12]. Abnormal expression of lncRNA OIP5-AS1 in thyroid cancer tissues suggests its potential of serving as a molecular biomarker for thyroid cancer [13].

As an important genetic variation, genome copy number variation (CNV) has specific changes in progression of certain cancers and has become an important characteristic of tumors. CNV refers to the submicroscopic mutation of DNA fragments from KB to Mb, mainly including replication, deletion, embedding, and complex multisite variation [14]. Redon et al. [15] filtered about 1500 CNVs regions in 270 samples, accounting for about 10% of the human genome and included coding and noncoding regions. In recent years, copy number variation has been found in tumors, and some studies have made breakthrough discoveries. For example, Ye et al. reported that medullary thyroid cancer is relevant to the changes in DNA copy number [16]. One study found that DNA copy number variation is a characteristic of malignant and benign thyroid tumors and that the segmentary expansion of chromosomes (Ch)7 and 12 is less common in follicular variant papillary thyroid cancers or typical papillary thyroid cancers than in follicular adenomas. In addition, the deletion in Ch22 has been detected in a subgroup of follicular adenoma and follicular variant thyroid papillary carcinoma [17]. However, the role of genome-level changes in the number of DNA copies in the pathogenesis of tumors is still controversial and can only explain the pathogenesis of certain patients [18], indicating that the mechanism of CNVs for tumor genesis is not well understood, especially the high-frequency genome variation in the desert region of the tumor, which is less studied. Therefore, the pathogenic mechanism of CNVs for abnormal expression of noncoding RNA, especially lnc RNA, requires further research.

Combining correlation analysis of CNV and lncRNA expression, we found that lncRNAs showing a transcriptional imbalance played a critical role in the biological process of thyroid cancer. We identified three CNV-related lncRNAs as prognostic biomarkers for predicting prognosis of thyroid cancer. Moreover, these prognostic lncRNAs could provide inspiration to discover novel molecular drugs for thyroid cancer treatment.

## 2. Materials and Methods

### 2.1. Large-Scale Data Selection

A total of 500 thyroid cancer samples were included in this study. 354 (70.8%) had thyroid papillary-classical/usual carcinoma, 101 cases (20.2%) had thyroid papillary carcinoma-follicular patterned (≥99.0%), 36 cases (7.2%) had thyroid papillary carcinoma-tall cell (≥50.0% tall cell features) type, and 9 cases (1.8%) were diagnosed with other unknown tissue type.

**Figure 1:**
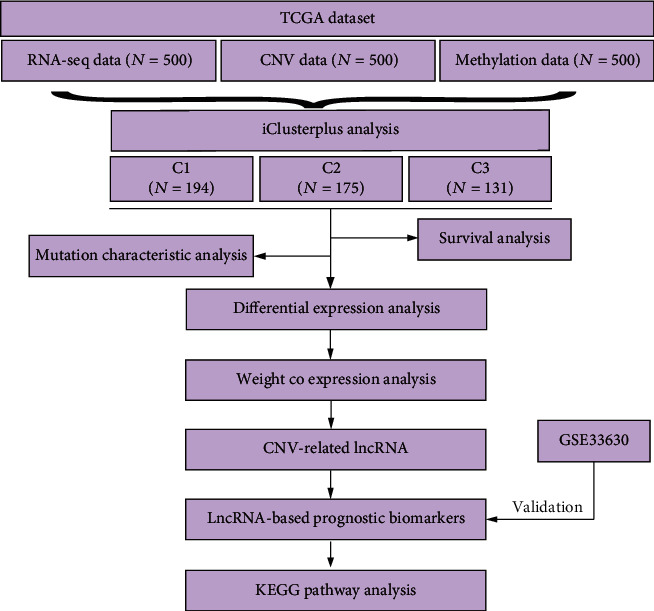
The flow chart of this study.

From the Cancer Genome Atlas (TCGA) Genomic Data Commons (GDC) database (https://gdc.cancer.gov/developers/gdc-application-programming-interface-api) CNV data, CpG data, RNA-seq data, clinical follow-up information, and mutation data of thyroid cancer were collected. Firstly, to process RNA-Seq data, counts data and all fragments per kilobase million (FPKM) data were obtained. FPKM was converted to transcripts per kilobase millions (TPM). Messenger RNA of lncRNA, 3prime_overlapping_ncRNA, sense_overlapping, antisense, processed_transcript, and sense_intronic were regarded as lncRNAs according to the gene types in genecode.v22.gtf file. In this way, the FPKM expression profile of lncRNAs was extracted. Also, the FPKM expression profiling of protein-coding genes (PCGs) was acquired. After obtaining the expression profile of all 450 k samples, CpG probes with NA in the expression of samples, CpG probes with cross-reactive site samples, and CpG probes on sex chromosomes and single nucleotide sites were removed from the CpG data. From TCGA GDC, the CNVs of all samples without type differences were downloaded. MuTect [19] software processed-single nucleotide mutation data were obtained from TCGA GDC (see Figure 1 for the flow chart of this study).

### 2.2. Univariate Survival Analysis

The influence of CNV, methylation, and coding genes on the cancer prognosis was analyzed independently to better classify the samples. We selected samples with a follow-up time longer than 30 days. Based on univariate COX proportional hazard regression (threshold: *P* = 0.05), a model was established. 3074 CNV regions, 40,150 CpG sites, and 1447 coding genes were finally screened.

### 2.3. Identification of Molecular Subtypes and Differential Analysis of lncRNA and PCGs

The CNV, prognostic coding genes and methylation sites were obtained by univariate survival analysis, and from the three common omics samples, a total of 500 samples were obtained. Multi-cluster analysis in R software package iClusterPlus [20] was conducted, and the number of classification was selected to be 3. Next, differentially expressed lncRNAs and PCGs in different subtypes were analyzed using R software package DEseq2 [21]. In the expression profile, the genes showing an average Count<1 were removed. In each subtype, the threshold of false discovery rate (FDR) <0.05 and fold change more than twice was applied to identify PCGs and the differentially expressed lncRNAs (DElncRNAs).

### 2.4. Weighted Gene Co-Expression Network Construction

To mine the co-expression module based on lncRNA expression profiles and differentially expressed PCGs, WGCNA co-expression algorithm in the R package WGCNA (http://www.r-project.org/) was employed [22]. By approximate scale-free topology criteria, we determined the soft threshold. Adjacency matrix was converted to topological matrix (TOM). Next, average-linkage hierarchical clustering was employed to cluster genes. Finally, we used dynamic tree cut method to determine module eigengenes with at least 30 co-expressed genes.

### 2.5. Identification of CNV-Driven lncRNAs

Visualization of regions in the genome by the Genomic Identification of Significant Targets in Cancer (GISTIC) [23] shows the deletion or amplification in thousands of samples. Thus, the copy number data of 505 TCGA thyroid cancer cases were analyzed by GISTIC 2.0 software. Firstly, the copy number spectrum of lncRNAs was developed. The copy number was amplified when >1, and a copy number deletion was defined when and the copy number ≤ -1. The ratio of amplification to deletion in the lncRNAs was determined, and the distribution in genome was observed.

### 2.6. Functional Pathway Enrichment Analysis in Module

The functions of hub genes in the module were examined. By loading “anRichment” package in R [20], the gene ontology (GO) enrichment analysis was conducted. *P* < 0.05 was the threshold.

### 2.7. Gene Set Enrichment Analysis

Gene set enrichment analysis (GSEA) [24] was performed and mapped into pathway enrichment database of Kyoto Encyclopedia of Genes and Genomes (KEGG) to study mechanisms of DElncRNAs in TCGA and GSE33630. Here, the cutoff was FDR < 0.05.

### 2.8. Statistical Analysis

Kaplan-Meier was used to plot survival curves using log-rank test. A significance was defined as *P* < 0.05. ROC analysis was conducted by using pROC R package [25]. Unless otherwise stated, all the analyses were carried out in R 3.4.3 under default parameters.

## 3. Results

### 3.1. Identification of Three Molecular Subtypes Based on Prognostic Methylation Sites, CNV, and PCGs

**Figure 2:**
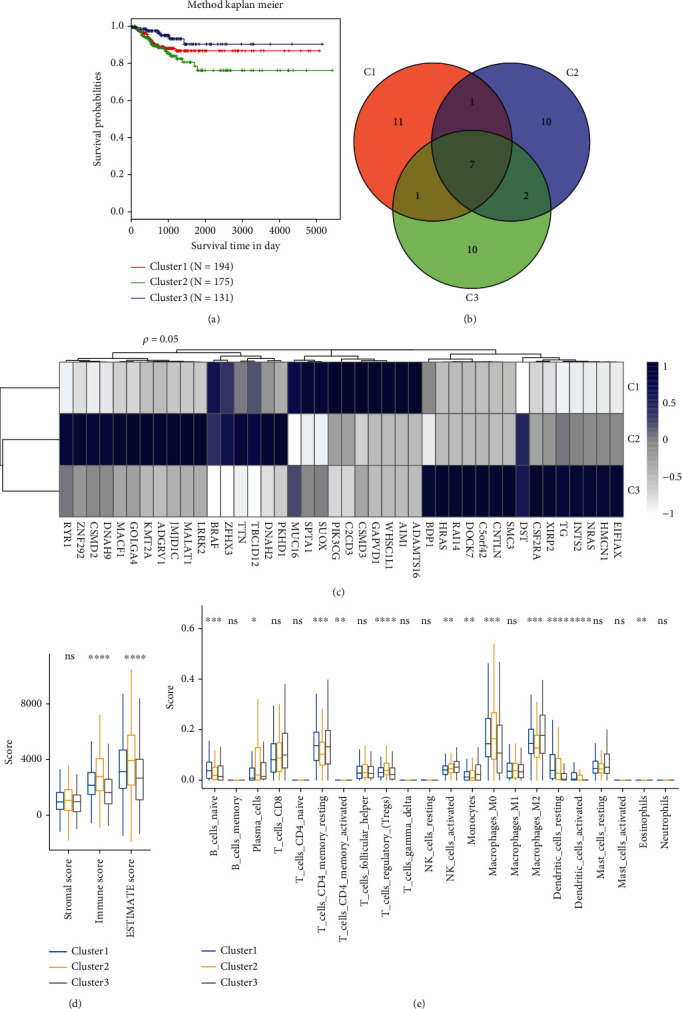
Molecular subtypes were identified by multi-omics analysis. (a) KM curves for disease-free survival of 3 molecular subtypes. (b) A Venn diagram for the top 20 genes showing the highest mutations in each molecular subtype. (c) Heat maps of the top 20 genes with the highest mutations in each molecular subtype. (d) The distribution of immune infiltration microenvironment scores of the three subtypes was different. (e) Distribution difference of three subtypes in 22 immune cell infiltration scores.

We identified differentially expressed CNV, methylation sites, and PCGs related to prognosis by comparing cancer samples to normal samples, and they were used as a basis to construct molecular subtypes using iClusterPlus. Three molecular subtypes were obtained (Table 1). From the Kaplan-Meier survival curve, obvious prognostic differences among the three molecular subtypes could be observed (Figure 2(a)). Furthermore, gene mutations in different subtypes were determined. In each subtype, 20 genes showing the highest mutation were selected, and a total of 42 genes were acquired. The intersection between the 20 genes with the highest mutation frequency was determined from the three subtype samples (Figure 2(b)). Also, the mutations of these 42 genes in various subtypes were visualized (Figure 2(c)). In addition, we used the R software package estimate to evaluate the immune microenvironment infiltration score of each patient. By analyzing the difference of the immune microenvironment infiltration scores of the three subtypes, we can observe that cluster2 has the significantly highest immune infiltration (Figure 2(d)). Using the R software package cibersort to evaluate the score of immune cell infiltration in 22, it can be observed that T_cells_regulatory_(Tregs) and Macrophages_M0 in cluster2 have the significantly highest infiltration score and cluster2 has the significantly lowest immune infiltration score in T_cells_CD4_memory_resting and Macrophages_M2. These results show that these three molecular subtypes have different immune microenvironment characteristics.

### 3.2. DElncRNAs and PCGs Were Identified from the Three Molecular Subtypes

**Figure 3:**
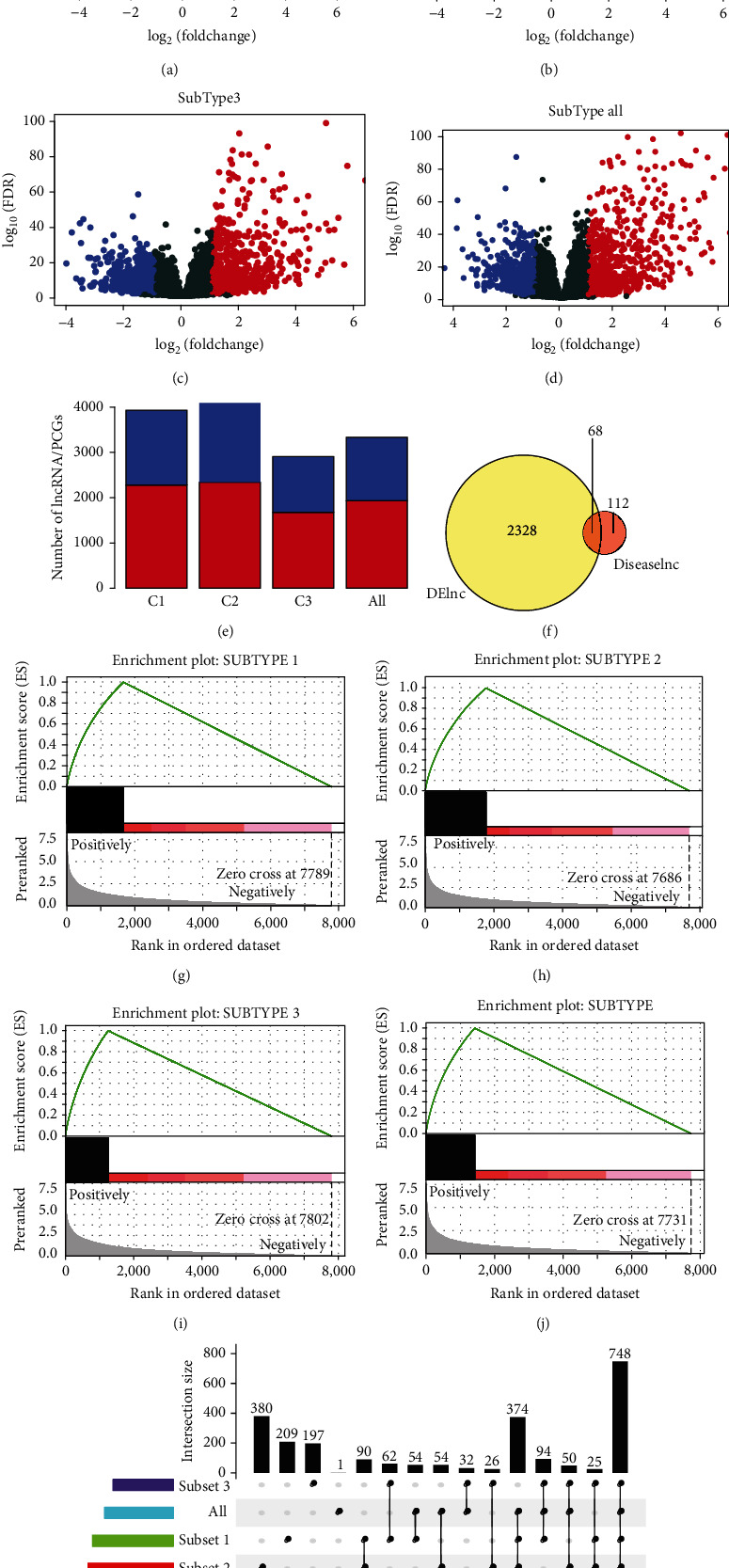
Identification of differentially expressed in lncRNAs and PCGs of different subtypes. (a–c) Volcano map of differentially expressed lncRNAs in three molecular subtypes, respectively. (d) Volcano map of differentially expressed lncRNAs in all subtypes. Red means up-expression and blue means down-expression. (e) Distribution of differential lncRNA and PCGs in molecular subtypes. (f) The Venn diagram of the differential lncRNA and disease-related lncRNA. *P* < 0.033. (g–j) GSEA analysis of three molecular subtypes as the rank according to the difference multiple. (k) The intersection of differentially expressed lncRNAs in three molecular subtypes.

We obtained 3254 PCGs and 2396 DElncRNAs in the molecular subtypes using DEseq2. (Table 2) and constructed a volcano map based on DElncRNAs screened from the three subtypes (Figures 3(a)–3(d)). In each subtype, the number of lncRNA and PCGs differences was counted, and we found that lncRNAs differences were generally smaller than PCGs differences (Figure 3(e)). Also, lncRNAs associated with the cancer were downloaded from Lnc2Cancer and LncRNADisease. 611 lncRNAs were acquired and compared with 2396 lncRNAs in different subtypes. There are 68 shared lncRNAs (Figure 3(f)). Furthermore, we performed GSEA analysis according to the absolute value of the difference multiple of lncRNA in each subtype as the rank. The data indicated that the differentially expressed lncRNA was clustered in the gene concentration with large difference multiple (Figures 3(g)–3(j)). Finally, between the tumorous and healthy samples, the intersection of the three subtypes and DElncRNAs was analyzed; here, the two showed a large intersection (Figure 3(k)).

### 3.3. Identification of Co-Expression Modules Base on Differentially Expressed PCGs and lncRNAs

**Figure 4:**
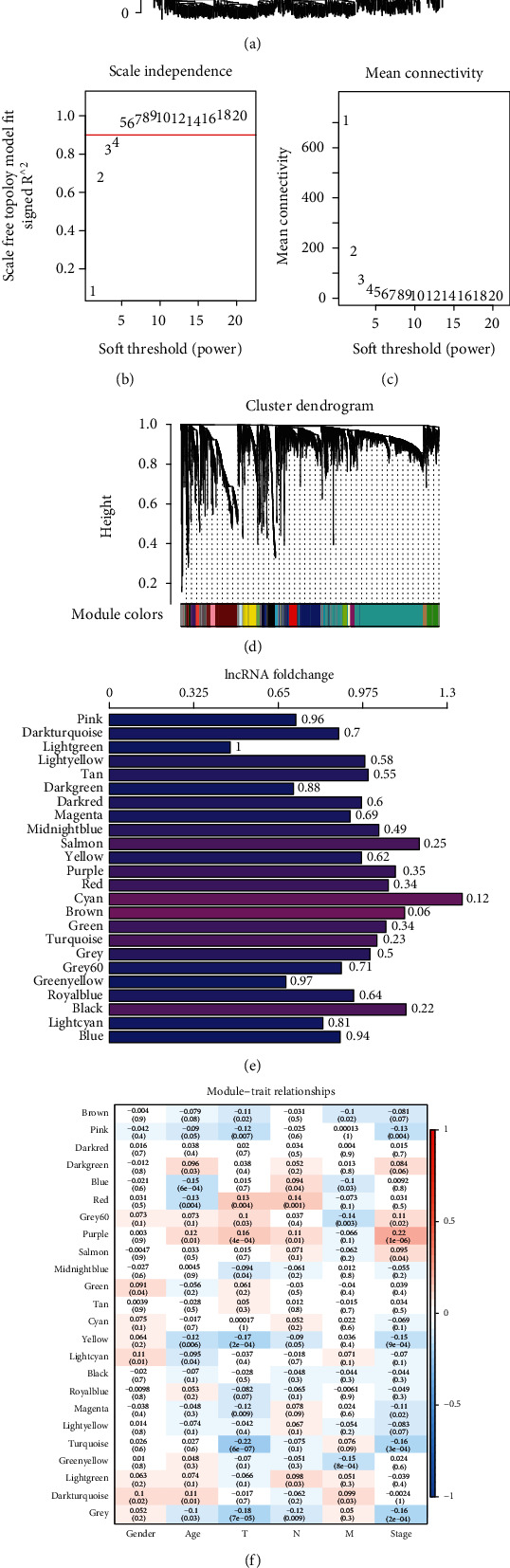
Identification of co-expression modules based on differentially expressed PCGs and lncRNAs. (a) Cluster analysis on the samples. (b–c) Network topology analysis for different soft-thresholding powers. (d) Module colors and gene dendrogram. (e) Histogram of relative multiples of lncRNA ratio to PCG ratio in 23 modules. The horizontal axis is the multiple of the lncRNA ratio to the PCG ratio in the module, vertical axis is module, and the values on the right are significant *P* values. (f) Correlation between 24 modules and clinical phenotype.

Firstly, hierarchical clustering on the samples was performed. Samples with a distance of more than 100,000 were screened as outliers, and 560 samples were obtained (Figure 4(a)). The co-expression network conformed to the scale-free network, as shown by the current data. To further ensure a scale-free nature of the network, *ꞵ* = 4 was selected (Figures 4(b) and 4(c)). R package WGCNA showed 24 modules (Figure 4(d), Table 3). Note that the grey module cannot be aggregated into other modules. In 23 modules, the histogram of relative multiples of lncRNA ratio to PCG showed no significant difference between lncRNA and genes in each module (Figure 4(e)). Further, the relationship of clinical status with modules was analyzed, and the modules are related to at least one phenotype, except dark red, cyan, tan, black, royal blue, and light yellow (Figure 4(f)). Modules related to at least three phenotypes were selected, yellow and purple, for subsequent analysis. Next, the unctional enrichment analysis was conducted on yellow and purple modules. In the yellow module, the top 20 GO terms enriched were mainly associated with cell proliferation and vascular system (Figure S1A). The top 20 pathways were mainly related to ligand-receptor interaction, PI3K−Akt, MAPK, and other pathways (Figure S1B). In the purple module, the top 20 GO terms enriched were mainly correlated with extracellular matrix and cell differentiation (Figure S1C). Among the top 20 pathways enriched, the most significant pathways were ECM-receptor interaction, PI3K-Akt, and Wnt signaling pathways (Figure S1D).

### 3.4. Identification of lncRNAs Driven by CNV

**Figure 5:**
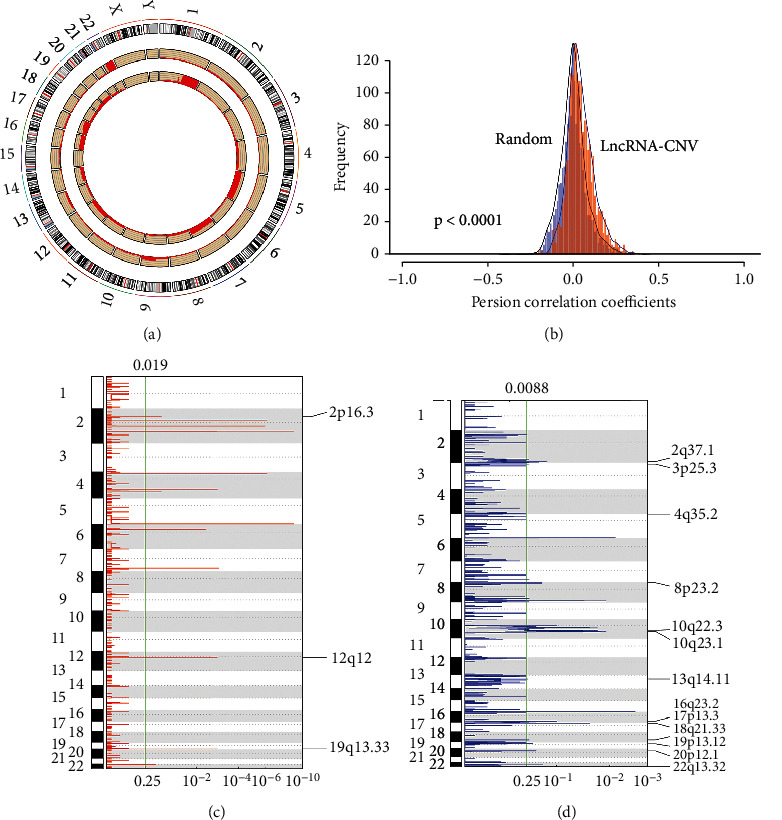
Identification of lncRNAs driven by CNV. (a) Distribution of lncRNA copy deletions and amplification in the genome. (b) Correlation distribution of CNV and expression of lncRNAs: light red represents the distribution under actual conditions, light blue represents the distribution under random conditions, and the difference between the two distributions is assessed by the T-test. (c–d) LincRNAs located in the focal CNA peaks are THCA-related. False-discovery rates (*q* values) and scores from GISTIC 2.0 for alterations (*x*-axis) are plotted against genome positions (*y*-axis); the dotted lines show the centromeres. The deletions (right and blue) and amplifications (left and red) of lincRNA genes are presented. The green line shows 0.25 *q* value cutoff point that determines significance.

**Figure 6:**
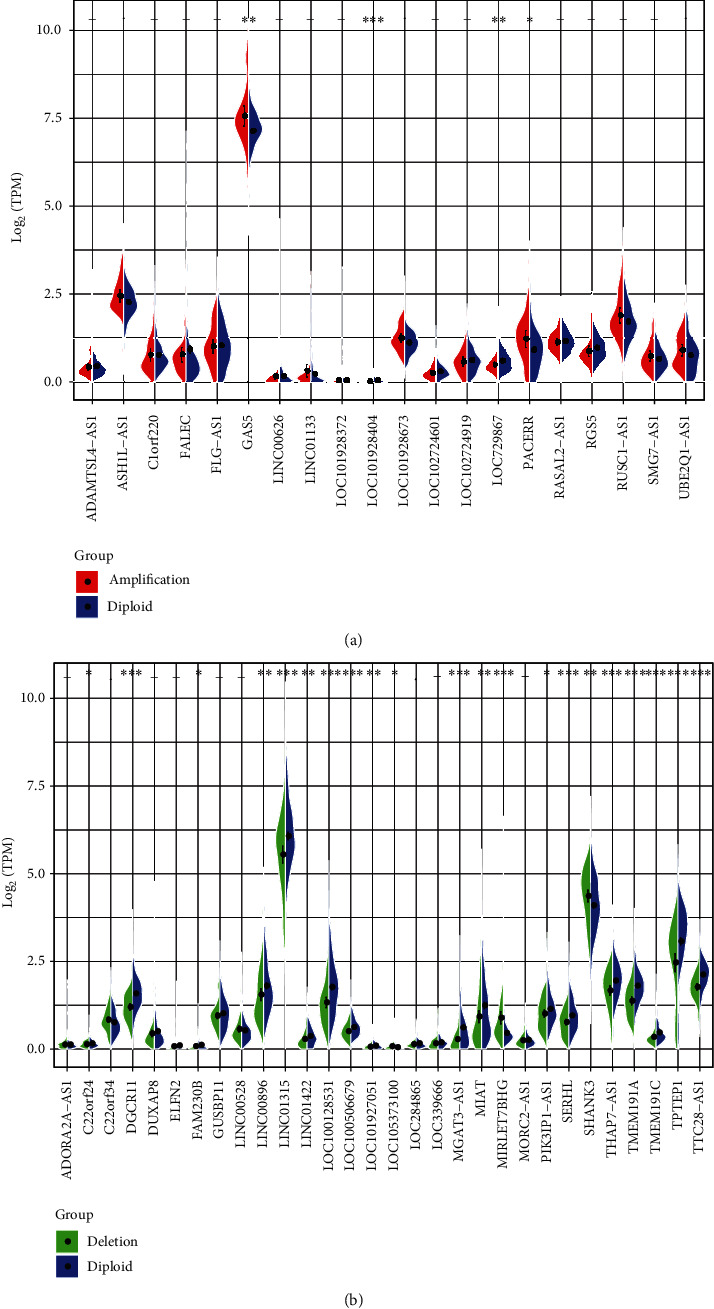
Different expression of lncRNA in copy amplified or deleted and normal copied samples. (a) 20 lncRNAs were copy-amplified, most of which were highly expressed in the copy-amplified samples. (b) 30 lncRNAs were copy-deleted, and the vast majority of which were significantly lowly expressed in the copy-deleted samples.

A sum of 505 TCGA thyroid cancer copy number were analyzed by GISTIC. The distribution of copy numbers across the genome were observed and found that the proportion of copy number amplification on chromosomes 1, 7, 12, 12, 16, and 17 was larger and the copy number deletion was the highest on chromosomes 9, 13, and 22 (Figure 5(a)). Furthermore, the correlation distribution of copy number and lncRNA expression profile was calculated. An overall positive correlation trend of the copy number and lncRNA expression and a significantly higher distribution than random were observed (Figure 5(b)). We identified frequently changing regions in the genome of thyroid cancer. Many lncRNAs showed significant multiple copies or significant deletions, while compared with copy amplification regions, lncRNA deletions were more frequent (Figures 5(c) and 5(d)). To further reveal the correlation of copy number with lncRNA expression, in each sample, 119 lncRNAs showing a copy number ratio of more than 7% were selected. Differences in expression of lncRNAs in the copy-amplified or copy-deficient samples as well as in the samples with normal copies were explored. LncRNAs whose expression was greater than 0 in at least 50% of the samples were selected, and finally 50 lncRNAs that met the conditions were retained. Among them, 20 lncRNAs were copy-amplified (Figure 6(a)), most of which were high-expressed in the copy-amplified samples. 30 lncRNAs were copy-deleted (Figure 6(b)), a great majority of which were significantly low-expressed in the copy-deleted samples. These results suggested that lncRNA copy changes were positively correlated with lncRNA expression.

### 3.5. Three lncRNAs Were Associated with Prognostic in Thyroid Cancer Were Determined

In order to systematically identify the prognostic markers of lncRNA, the criteria for inclusion were that lncRNAs showing a copy number change of more than 0.1% in each sample, differential lncRNAs in at least three subtypes and positive correlation of expression of lncRNAs with the copy number. Here, 8 lncRNAs significantly associated with prognosis were screened (Table 4).

**Figure 7:**
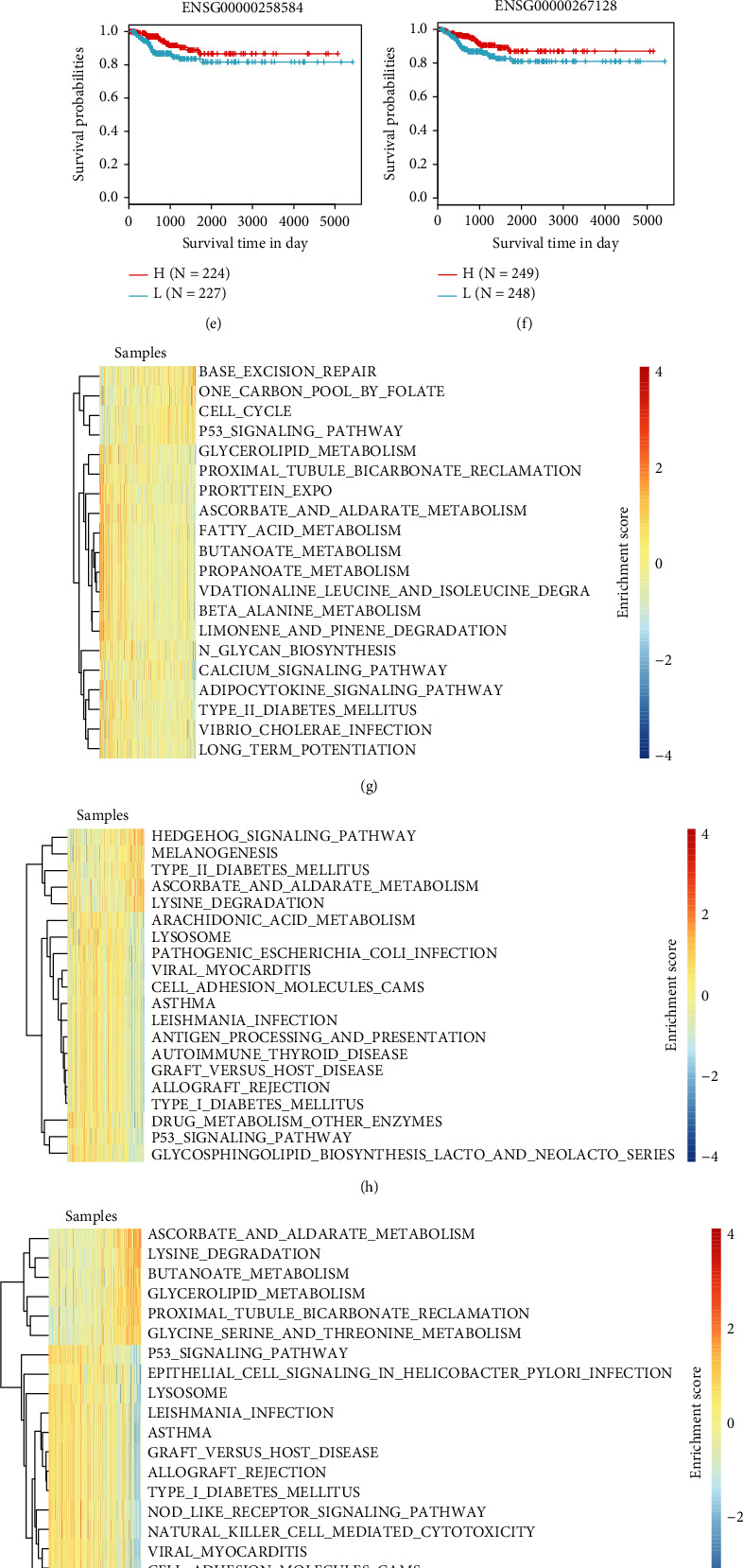
Three lncRNAs were associated with prognosis in thyroid cancer. (a–c) ROC curve of FOXD2-AS1, FAM181A-AS1, and RNF157-AS1 in the TCGA dataset. (d–f) KM survival curve of FOXD2-AS1, FAM181A-AS1, and RNF157-AS1 in the TCGA dataset. (g–i) KEGG pathway analysis of FOXD2-AS1, FAM181A-AS1, and RNF157-AS1 in the TCGA dataset. (j) The expression and distribution of three lncrnas were different in three molecular subtypes.

Further, the effectiveness of the 8 lncRNAs in prognostic classification was analyzed, according to their expression in each sample, and then we screened a total of 3 lncRNAs with AUC greater than 0.55 (Figures 7(a)–7(c)). KM survival curve showed that only FOXD2-AS1 neatly grouped the samples into two risk groups (high, low) (Figures 7(d)–7(f)). ssGSEA was used to perform enrichment analysis on KEGG pathway of each sample and screened the top 20 KEGG pathway showing the highest correlation with lncRNA expression. The top 20 KEGG pathway correlation coefficients of the three lncRNAs were higher than 0.23, 0.34, and 0.47, respectively (Figures 7(g)–7(i)). There were 4 positive correlations with FOXD2-AS1, including P53 signaling pathway, cell cycle, and other cancer-related pathways, and 16 negative correlation pathways mainly related to metabolism. In FAM181A-AS1 and RNF157-AS1 and P53 signaling pathway, cell adhesion molecules functioning similar to cancer-related pathways were all negatively correlated. In addition, there were significant differences in the expression of the three lncrnas among the three subtypes. ENSG00000267128 (RNF157-AS1) and ENSG00000258584 (FAM181A-AS1) were significantly overexpressed in cluster3, and ENSG00000237424 (FOXD2-AS1) was significantly overexpressed in cluster1 (Figure 7(j)). In short, the expression of these three lncRNAs was greatly involved in the cancer initiation and progression.

### 3.6. Validation of Three lncRNAs in GSE33630

**Figure 8:**
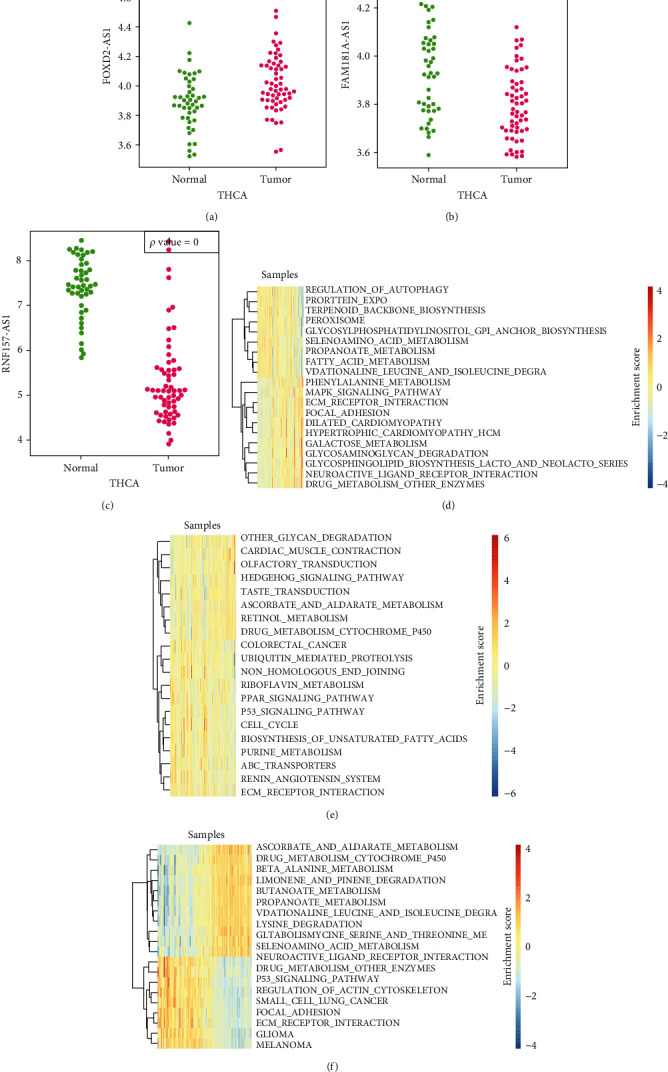
Three lncRNAs associated with prognosis in thyroid cancer were validated in GSE33630 dataset. (a–c) The expressions of FOXD2-AS1, FAM181A-AS1, and RNF157-AS1 between the tumor and normal in the GSE33630 dataset. (d–f) KEGG pathway analysis of FOXD2-AS1, FAM181A-AS1, and RNF157-AS1 in the GSE33630 dataset.

For further verifying the role of the three CNV-related lncRNAs in thyroid cancer, a dataset GSE33630 including 60 thyroid samples and 45 normal samples was downloaded from GEO. The reannotation data revealed a significantly high expression of FOXD2-AS1 in thyroid cancer samples (Figure 8(a)). FAM181A-AS1 and NF157-AS1 showed a low expression in tumor samples (Figures 8(b)–8(c)). Meanwhile, functional enrichment analysis demonstrated that the correlation coefficients of the top 20 KEGG pathway were above 0.36, 0.24 and 0.71, respectively (Figures 8(d)–8(f)). Among them, FOXD2-AS1 was positively correlated with 11 pathways, including ECM receptor interaction, MAPK signaling pathway, and some other cancer-related pathways, and negatively correlated with 9 pathways, mainly including REGULATION_OF_AUTOPHAGY and metabolism-related pathways. In FAM181A-AS1 and RNF157-AS1, P53 signaling pathway and ECM receptor interaction were negatively correlated with cancer-related pathways, which further indicated that the expression levels of the three lncRNAs were closely associated with cancer initiation and progression.

### 3.7. Verification of the Relationship between the Three lncRNAs and Prognosis

The overall survival rate of thyroid cancer was noticeably high, and follow-up to the end of the event was more than 10 years. Therefore, the cohort data with survival information are rare at present. We also evaluated the relationship between the lncRNAs and prognosis from the clinical characteristics of the samples. We obtained a gene chip dataset GSE60542 from the GEO database, matched the probe to the gene, extracted the expression profile of the three lncRNAs, and evaluated the expression difference of the three lncRNAs in the early and late samples. FOXD2-AS1 was found to be significantly overexpressed in the late samples, and FAM181A-AS1 was significantly overexpressed in the early samples (Figure S2A). In addition, a significant negative correlation between FAM181A-AS1 and lymph node metastasis was observed (Figure S2B). These results suggested that FOXD2-AS1 was a risk factor and FAM181A-AS1 was a protective factor.

Furthermore, a set of TCGA sample exon quantification dataset (https://xenabrowser.net/datapages/?dataset=TCGA.THCA.sampleMap%2FHiSeqV2_exon&host=https%3A%2F%2Ftcga.xenahubs.net&removeHub=https%3A%2F%2Fxena.treehouse.gi.ucsc.edu%3A443) was supplemented. The average expression level of the three lncRNA exons of each sample was evaluated. Among them, only FAM181A-AS1 and RNF157-AS1 were detected; therefore, the correlation of the prognosis with expression of these two lncRNAs was verified. The optimal truncation value of each lncRNA expression was obtained using Maxstat, R software package. We observed that the low expression group was associated with poor prognosis (Figure S2C), which was consistent with previous results.

## 4. Discussion

In this study, we provided a comprehensive set of dysregulated lncRNAs driven by CNV in thyroid cancer. Three molecular subtypes with differential prognosis of thyroid cancer patients were categorized using multi-omic data. These three subtypes have significantly different molecular characteristics and prognosis. Cluster2 patients have the highest immune microenvironment score, and cluster2 has the lowest immune microenvironment score. These differences may lead to different benefits in immunotherapy and may also be an important reason for the different prognosis of these patients. 2396 DElncRNAs from the molecular subtypes were obtained. There were many regions in the thyroid cancer genome with multiple copies or significant deletions of lncRNAs. Furthermore, three CNV-driven lncRNAs showed a significant correlation to overall survival, and the dysregulated lncRNAs driven by CNV were involved in some critical biological functions in thyroid cancer, such as cell cycle, metabolism pathways, and P53 signaling pathway, indicating that they may be potential clinical biomarkers of prognosis.

As a common structural variation in human genome, CNV has been regarded as an important contributing factor to caner development [26, 27]. Panebianco et al. found a higher TERT promoter CNV in advanced thyroid cancer [28]. Five CNV-related genes (VEZT, NDUFA12, GDF3, FGD6, and NR2C1) have been identified as the most effective genes to distinguish FAs from PTCs/FVPTCs, as they could correctly classify 90% of the cases [17]. CNV loss on the FMN2 gene promotes lymph node metastasis in medullary thyroid carcinoma family [29]. However, no studies have been performed on lncRNA CVA in thyroid cancer. In ovarian cancer prognosis-correlated, lncRNAs as novel biomolecular markers have been discovered for based CNV analysis [30]. In this study, for the first time, the prognostic indicators of thyroid cancer were identified based on lncRNA CNV. CNV-driven lncRNAs may contribute to thyroid cancer development and have the potential of serving as biomarker for thyroid cancer diagnosis.

Three copy number-related lncRNAs (FOXD2-AS1, FAM181A-AS1, and RNF157-AS1) significantly correlated with thyroid cancer survival were identified. FOXD2-AS1 was high-expressed in thyroid cancer tissues and cell lines, and this was related to tumor progression and metastasis [31–33]. This was consistent with our findings that high-expressed FOXD2-AS1 could predict a poor survival and that tumor related pathways such as P53 signaling pathway and cell cycle were mainly the KEGG enrichment pathways of target genes regulated by FOXD2-AS1, indicating that the analysis of whole genome lncRNA CNV to identify prognosis of thyroid cancer was an effective and reliable approach. However, no previous study investigated FAM181A-AS1 and RNF157-AS1. Based on the current literature review and the current findings, we hypothesized that CNV caused changes in the three lncRNA expressions and affected the progression of thyroid cancer through the regulatory mechanism of ceRNA. We inversely searched the miRNAs targeting these three lncRNAs from the Starbase database and observed that there were 16 miRNAs targeting ENSG000000237424 (FOXD2-AS1). Further, we matched the protein coding genes targeted by these 16 miRNAs. According to the Cerna hypothesis, we constructed a statistical model to evaluate the significance of the competition between lncRNA-mRNAs and selected FDR<0.01 to obtain 50 genes, Further, we also screened lncRNA-mRNA with significant positive correlation and finally obtained 18 ceRNAs (Figure S2D). It can be observed that FOXD2-AS1 and TEX22 share the most miRNAs and high positive correlation. Those data suggested that the three lncRNAs may be important marker for thyroid cancer. Based on the above analysis, FAM181A-AS1 and RNF157-AS1 showed potential to help further reveal the mechanisms of thyroid cancer.

However, our study still has the following limitations. Clinical data and large datasets on thyroid cancer were insufficient. A lack of basic research was another limitation. Though the present analysis may not be the optimal, it should be sufficient to conclude that CNV leading to lncRNA dysregulation contributed to a poor prognosis of thyroid cancer. Basic experimental research and more in-depth functional research will be our future research direction.

## 5. Conclusions

At present, no studies have reported the relation of thyroid cancer with changes of lncRNA CNV. This study opened a new methodology to discover prognostic biomarkers for thyroid cancer through combining lncRNA expression profiles with CNV data. FOXD2-AS1, FAM181A-AS1, and RNF157-AS1 with abnormal CNV showed a difference on thyroid cancer survival and may promote the cancer development. Overall, this study identified three lncRNAs that could serve as prognostic biomarkers and provided a new insight into the possible mechanism of thyroid cancer development.

## Figures and Tables

**Table 1:** Number of samples of three subtypes.

Cluster	Sample count
C1	194
C2	175
C3	131

**Table 2:** Differential expression of PCG and lncRNA.

Type	C1	C2	C3	All
PCG_Down	1290	1165	817	898
PCG_Up	988	1179	859	1032
PCG_All	2278	2344	1676	1930
Lnc_Down	922	887	614	667
Lnc_Up	734	860	620	740
Lnc_All	1656	1747	1234	1407

**Table 3:** PCGs and lncRNA in modules.

Module	All	Lnc	PCG	*P* value	Fc
Blue	579	229	350	0.935	0.889
Light cyan	61	23	38	0.809	0.822
Black	162	74	88	0.219	1.142
Royal blue	44	18	26	0.636	0.940
Green yellow	90	30	60	0.970	0.679
Grey60	58	23	35	0.710	0.892
Grey	744	316	428	0.500	1.003
Turquoise	1832	790	1042	0.234	1.030
Green	239	105	134	0.336	1.064
Brown	562	256	306	0.061	1.136
Cyan	70	35	35	0.121	1.358
Red	188	83	105	0.338	1.074
Purple	105	47	58	0.346	1.101
Yellow	276	115	161	0.624	0.970
Salmon	77	36	41	0.253	1.192
Midnight blue	67	29	38	0.489	1.036
Magenta	106	43	63	0.685	0.927
Dark red	36	15	21	0.599	0.970
Dark green	35	12	23	0.875	0.709
Tan	78	33	45	0.551	0.996
Light yellow	50	21	29	0.577	0.983
Light green	51	13	38	0.996	0.465
Dark turquoise	33	13	20	0.699	0.883
Pink	107	37	70	0.961	0.718

**Table 4:** LncRNAs with significant prognosis.

Symbol	*P* value	HR	Low 95% CI	High 95% CI	CNV Rate	DECount	Pcc	GPL570 Probe
FOXD2-AS1	0.007	0.454	0.261	0.790	0.008	4	0.01	224457_at
PGM5P3-AS1	0.033	2.043	1.081	3.859	0.020	3	0.01	239287_at
LOC102724714	0.013	2.141	1.201	3.817	0.022	4	0.03	NA
HAND2-AS1	0.015	2.158	1.195	3.896	0.016	4	0.02	239708_at
LOC100507144	0.032	1.861	1.069	3.240	0.026	3	0.12	NA
FAM181A-AS1	0.037	1.895	1.056	3.400	0.026	3	0.08	1557211_a_at
FENDRR	0.017	2.033	1.168	3.540	0.044	3	0.06	243059_at
RNF157-AS1	0.046	1.784	1.025	3.106	0.051	3	0.10	230776_at

## Data Availability

The data used to support the findings of this study are included within the article.

## Supplementary Materials

Supplementary 1 Figure S1: functional enrichment analysis. (a) Top 20 GO term in the yellow module. (b) Top 20 KEGG pathways in the yellow module. (c) Top 20 GO term in the purple module. (d) Top 20 KEGG pathways in the purple module.

Supplementary 2 Figure S2: verification of the relationship between three lncRNAs and prognosis. (a) The expression differences of the three lncRNAs in the early and late samples in the GSE60542 dataset. (b) Correlation between FAM181A-AS1 expression and lymph node metastasis in GSE60542 dataset. (c) The KM curve of FAM181A-AS1 and RNF157-AS1 expression and prognosis in the TCGA exon dataset. (d) ceRNA network of 18 mRNAs associated with FOXD2-AS1.

## References

[1] Bray F., Ferlay J., Soerjomataram I., Siegel R. L., Torre L. A., Jemal A. (2018). Global cancer statistics 2018: GLOBOCAN estimates of incidence and mortality worldwide for 36 cancers in 185 countries. *CA: A Cancer Journal for Clinicians.*.

[2] Sherman S. I. (2003). Thyroid carcinoma. *The Lancet*.

[3] Hay I. D., Lee R. A., Davidge-Pitts C., Reading C. C., Charboneau J. W. (2013). Long-term outcome of ultrasound-guided percutaneous ethanol ablation of selected "recurrent" neck nodal metastases in 25 patients with TNM stages III or IVA papillary thyroid carcinoma previously treated by surgery and ^131^I therapy. *Surgery*.

[4] Hay I. D., Thompson G. B., Grant C. S. (2002). Papillary thyroid carcinoma managed at the Mayo Clinic during six decades (1940-1999): temporal trends in initial therapy and long-term outcome in 2444 consecutively treated patients. *World Journal of Surgery*.

[5] Yang Z. T., Li Z., Wang X. G. (2015). Overexpression of long non-coding RNA ZXF2 promotes lung adenocarcinoma progression through c-Myc pathway. *Cellular Physiology and Biochemistry*.

[6] Lin W., Zhou Q., Wang C. Q. (2020). LncRNAs regulate metabolism in cancer. *International Journal of Biological Sciences*.

[7] Jiang W., Xia J., Xie S. (2020). Long non-coding RNAs as a determinant of cancer drug resistance: towards the overcoming of chemoresistance via modulation of lncRNAs. *Drug Resistance Updates*.

[8] Piao H. Y., Guo S., Wang Y., Zhang J. (2020). Exosomal long non-coding RNA CEBPA-AS1 inhibits tumor apoptosis and functions as a non-invasive biomarker for diagnosis of gastric cancer. *Onco Targets and Therapy*.

[9] Zhang Y., Zhang X., Zhu H. (2020). Identification of potential prognostic long non-coding RNA biomarkers for predicting recurrence in patients with cervical cancer. *Cancer Management and Research*.

[10] Xu M., Guo X., Wang R. D., Zhang Z. H., Jia Y. M., Sun X. (2020). Long non-coding RNA HANR as a biomarker for the diagnosis and prognosis of colorectal cancer. *Medicine*.

[11] Verhaegh G. W., Verkleij L., Vermeulen S. H., den Heijer M., Witjes J. A., Kiemeney L. A. (2008). Polymorphisms in the H19 gene and the risk of bladder cancer. *European Urology*.

[12] Liu L., Yang J., Zhu X., Li D., Lv Z., Zhang X. (2016). Long noncoding RNA H19 competitively binds miR-17-5p to regulate YES1 expression in thyroid cancer. *The FEBS Journal*.

[13] Li Q., Chen W., Luo R. (2020). Upregulation of OIP5-AS1 predicts poor prognosis and contributes to thyroid cancer cell proliferation and migration. *Molecular Therapy Nucleic Acids*.

[14] Wu D., Zhao J., Ma H., Wang M. C. (2020). Integrating transcriptome-wide association study and copy number variation study identifies candidate genes and pathways for diffuse non-Hodgkin's lymphoma. *Cancer Genetics*.

[15] Redon R., Ishikawa S., Fitch K. R. (2006). Global variation in copy number in the human genome. *Nature*.

[16] Ye L., Santarpia L., Cote G. J., El-Naggar A. K., Gagel R. F. (2008). High resolution array-comparative genomic hybridization profiling reveals deoxyribonucleic acid copy number alterations associated with medullary thyroid carcinoma. *The Journal of Clinical Endocrinology and Metabolism*.

[17] Liu Y., Cope L., Sun W. (2013). DNA copy number variations characterize benign and malignant thyroid tumors. *The Journal of Clinical Endocrinology and Metabolism*.

[18] Pollack J. R., Perou C. M., Alizadeh A. A. (1999). Genome-wide analysis of DNA copy-number changes using cDNA microarrays. *Nature Genetics*.

[19] Cibulskis K., Lawrence M. S., Carter S. L. (2013). Sensitive detection of somatic point mutations in impure and heterogeneous cancer samples. *Nature Biotechnology*.

[20] Yu G., Wang L. G., Han Y., He Q. Y. (2012). clusterProfiler: an R package for comparing biological themes among gene clusters. *Omics: a journal of Integrative Biology*.

[21] Love M. I., Huber W., Anders S. (2014). Moderated estimation of fold change and dispersion for RNA-seq data with DESeq2. *Genome Biology*.

[22] Langfelder P., Horvath S. (2008). WGCNA: an R package for weighted correlation network analysis. *BMC Bioinformatics*.

[23] Mermel C. H., Schumacher S. E., Hill B., Meyerson M. L., Beroukhim R., Getz G. (2011). GISTIC2.0 facilitates sensitive and confident localization of the targets of focal somatic copy-number alteration in human cancers. *Genome Biology*.

[24] Subramanian A., Kuehn H., Gould J., Tamayo P., Mesirov J. P. (2007). GSEA-P: a desktop application for gene set enrichment analysis. *Bioinformatics*.

[25] Robin X., Turck N., Hainard A. (2011). pROC: an open-source package for R and S+ to analyze and compare ROC curves. *BMC Bioinformatics*.

[26] Soong D., Stratford J., Avet-Loiseau H. (2020). CNV radar: an improved method for somatic copy number alteration characterization in oncology. *BMC Bioinformatics*.

[27] Alomari A. K., Miedema J. R., Carter M. D. (2020). DNA copy number changes correlate with clinical behavior in melanocytic neoplasms: proposal of an algorithmic approach. *Modern Pathology*.

[28] Panebianco F., Nikitski A. V., Nikiforova M. N., Nikiforov Y. E. (2019). Spectrum of TERT promoter mutations and mechanisms of activation in thyroid cancer. *Cancer Medicine*.

[29] Araujo A. N., Moraes L., França M. I. C. (2014). Genome-wide copy number analysis in a family with p.G533C RET mutation and medullary thyroid carcinoma identified regions potentially associated with a higher predisposition to lymph node metastasis. *The Journal of Clinical Endocrinology and Metabolism*.

[30] Zheng M., Hu Y., Gou R. (2020). Identification three LncRNA prognostic signature of ovarian cancer based on genome-wide copy number variation. *Biomedicine & Pharmacotherapy = Biomedecine & pharmacotherapie*.

[31] Li H., Han Q., Chen Y. (2019). Upregulation of the long non-coding RNA FOXD2-AS1 is correlated with tumor progression and metastasis in papillary thyroid cancer. *American Journal of Translational Research*.

[32] Zhang Y., Hu J., Zhou W., Gao H. (2019). LncRNA FOXD2-AS1 accelerates the papillary thyroid cancer progression through regulating the miR-485-5p/KLK7 axis. *Journal of Cellular Biochemistry*.

[33] Liu X., Fu Q., Li S. (2019). LncRNA FOXD2-AS1 functions AS a competing endogenous RNA to regulate TERT expression by sponging miR-7-5p in thyroid cancer. *Frontiers in Endocrinology*.

