# Supplementary figures and images for: Identification of Three Prognosis-Related Differentially Expressed lncRNAs Driven by Copy Number Variation in Thyroid Cancer
Source: J Immunol Res. 2022 May 20;2022:9203796. doi: 10.1155/2022/9203796 (PMC9148411; doi:10.1155/2022/9203796)

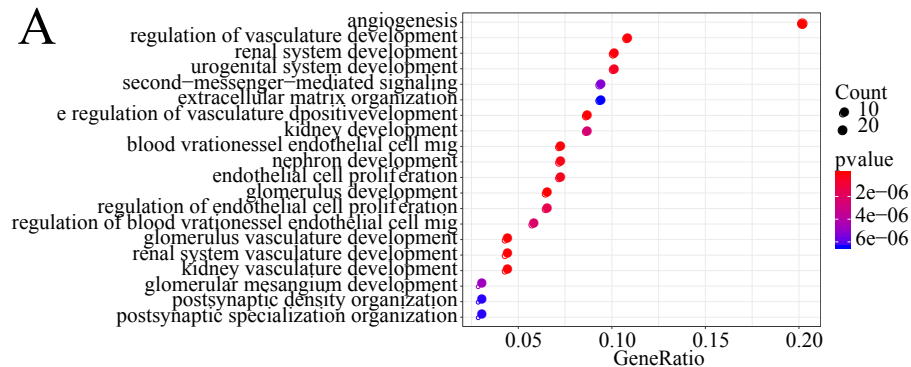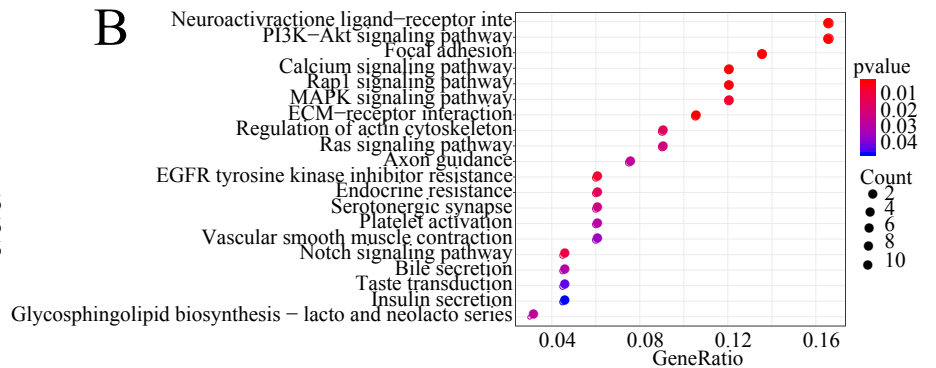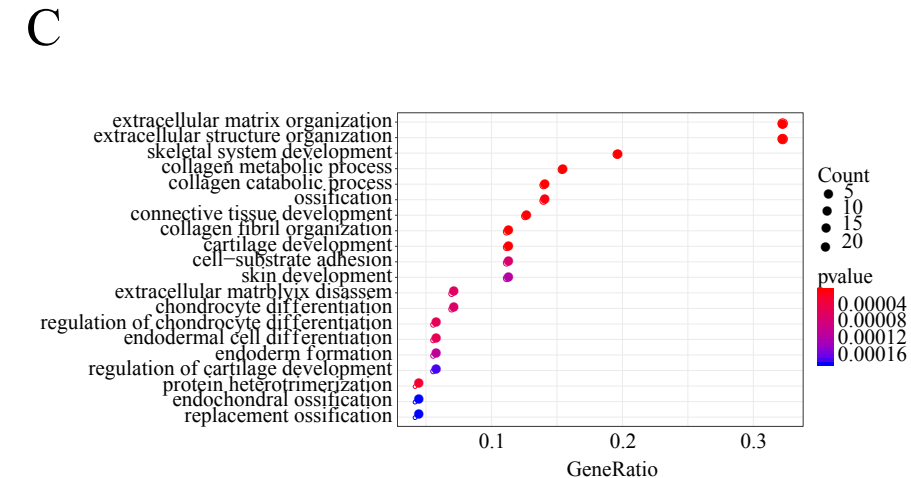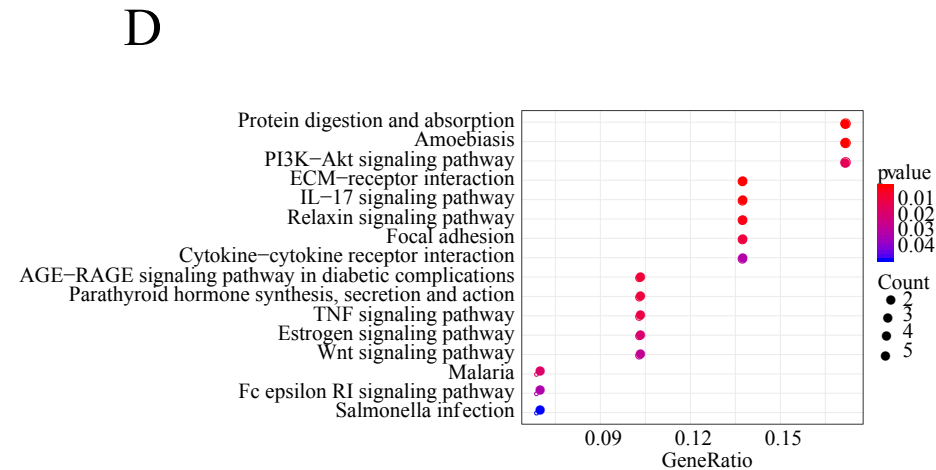

Supplement: Supplementary 1 — Figure S1: functional enrichment analysis. (a) Top 20 GO term in the yellow module. (b) Top 20 KEGG pathways in the yellow module. (c) Top 20 GO term in the purple module. (d) Top 20 KEGG pathways in the purple module. [file 9203796.f1.pdf]

A

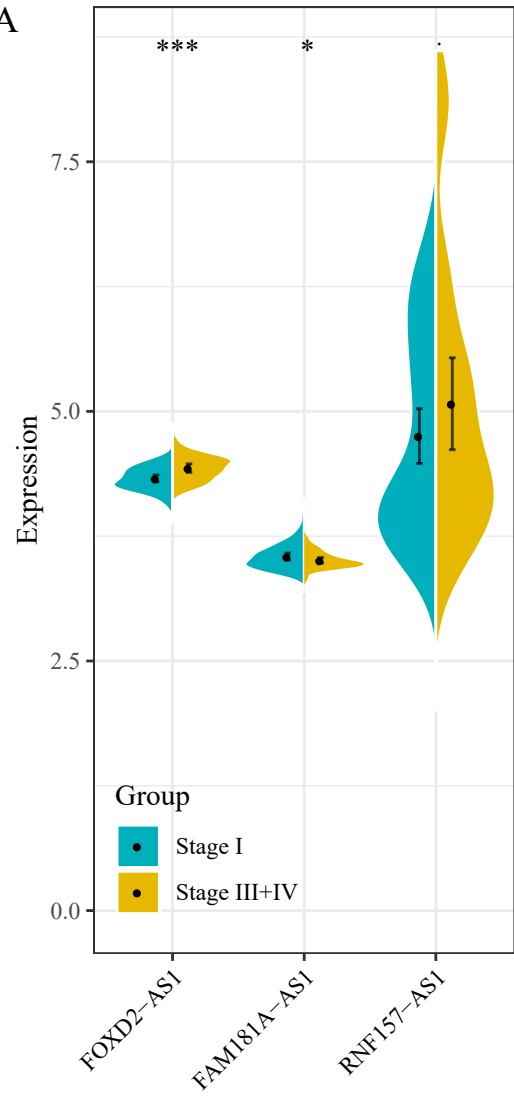

B

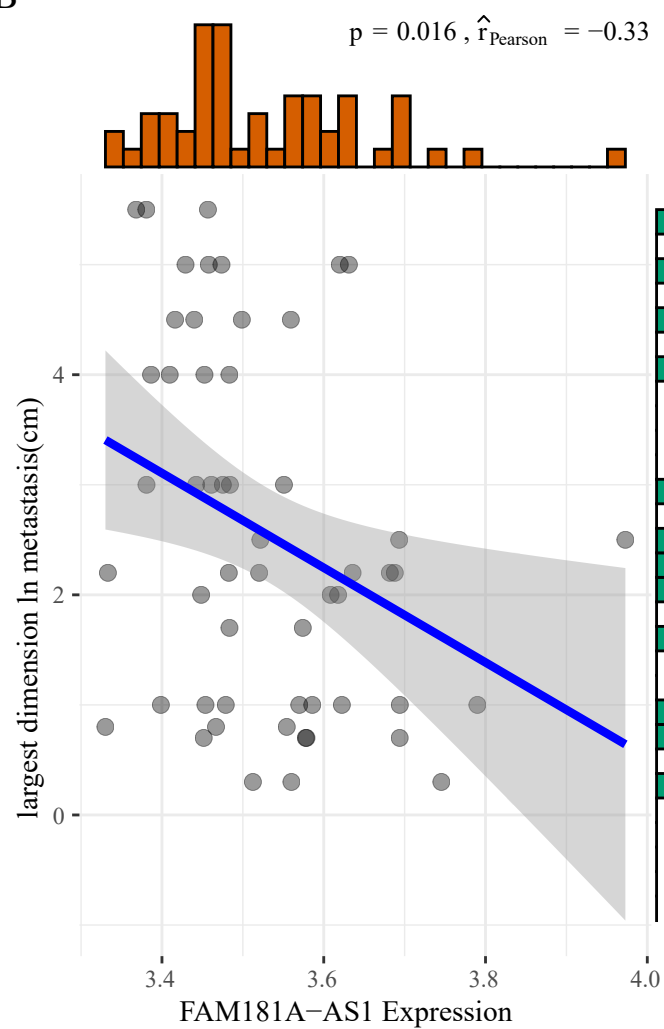

D

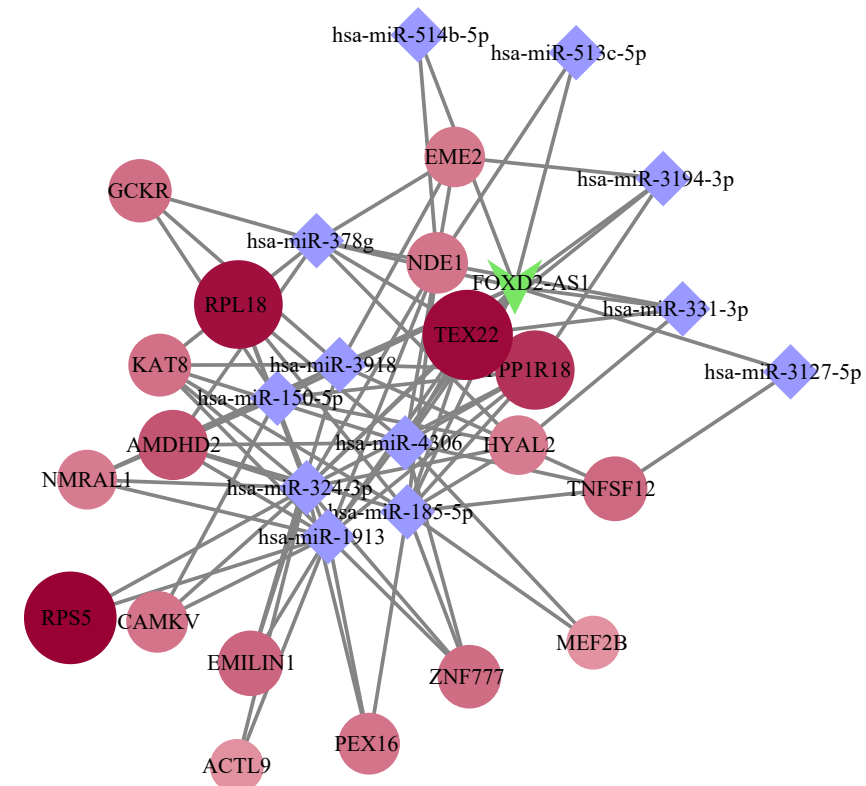

C

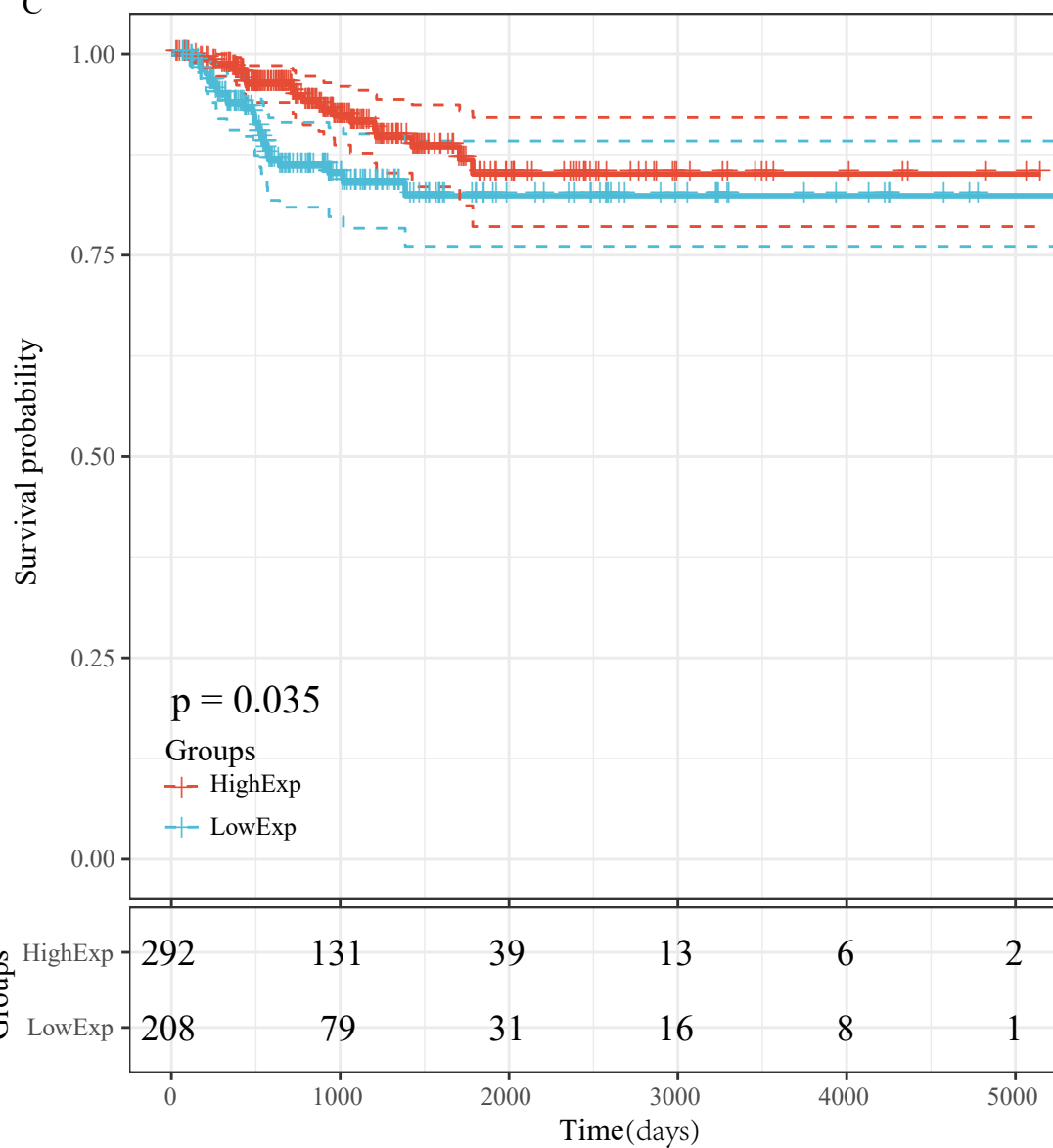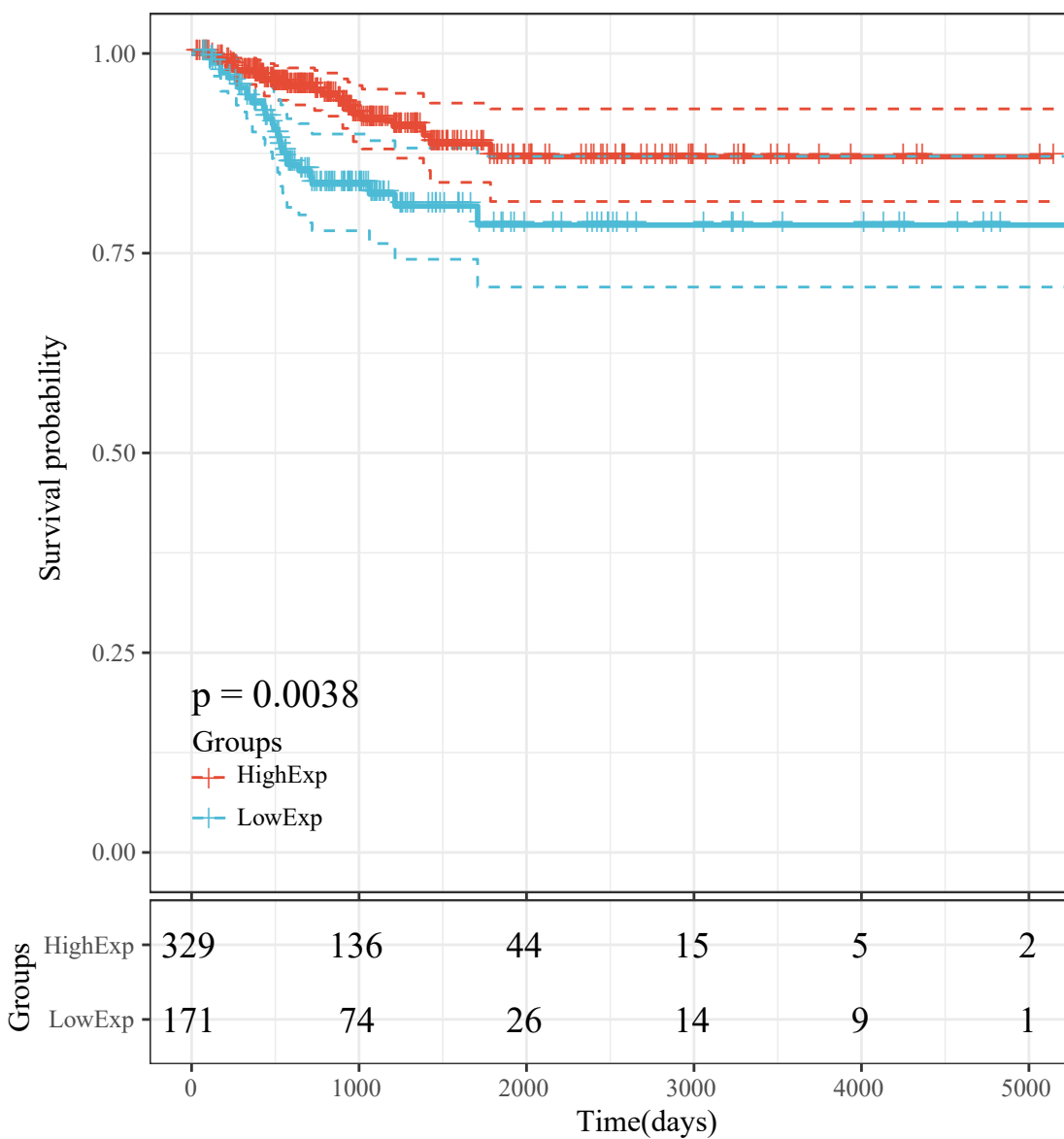

Supplement: Supplementary 2 — Figure S2: verification of the relationship between three lncRNAs and prognosis. (a) The expression differences of the three lncRNAs in the early and late samples in the GSE60542 dataset. (b) Correlation between FAM181A-AS1 expression and lymph node metastasis in GSE60542 dataset. (c) The KM curve of FAM181A-AS1 and RNF157-AS1 expression and prognosis in the TCGA exon dataset. (d) ceRNA network of 18 mRNAs associated with FOXD2-AS1. [file 9203796.f2.pdf]
